# The differential expression patterns of paralogs in response to stresses indicate expression and sequence divergences

**DOI:** 10.1186/s12870-020-02460-x

**Published:** 2020-06-16

**Authors:** Shuaibin Lian, Yongjie Zhou, Zixiao Liu, Andong Gong, Lin Cheng

**Affiliations:** 1 College of Physics and Electronic Engineering, Xinyang Normal University, Xinyang, China; 2 College of Life Sciences, Xinyang Normal University, Xinyang, China

**Keywords:** Paralogous gene pair, Differentially expressed gene, Whole genome duplication

## Abstract

**Background:**

Theoretically, paralogous genes generated through whole genome duplications should share identical expression levels due to their identical sequences and chromatin environments. However, functional divergences and expression differences have arisen due to selective pressures throughout evolution. A comprehensive investigation of the expression patterns of paralogous gene pairs in response to various stresses and a study of correlations between the expression levels and sequence divergences of the paralogs are needed.

**Results:**

In this study, we analyzed the expression patterns of paralogous genes under different types of stress and investigated the correlations between the expression levels and sequence divergences of the paralogs. We analyzed the differential expression patterns of the paralogs under four different types of stress (drought, cold, infection, and herbivory) and classified them into three main types according to their expression patterns. We then further analyzed the differential expression patterns under various degrees of stress and constructed corresponding co-expression networks of differentially expressed paralogs and transcription factors. Finally, we investigated the correlations between the expression levels and sequence divergences of the paralogs and identified positive correlations between expression level and sequence divergence. With regard to sequence divergence, we identified correlations between selective pressures and phylogenetic relationships.

**Conclusions:**

These results shed light on differential expression patterns of paralogs in response to environmental stresses and are helpful for understanding the relationships between expression levels and sequences divergences.

## Background

Several studies have found that most plants have undergone multiple rounds of whole genome duplication (WGD) [1–3], which has long been recognized as an important evolutionary force. At least one ancient WGD occurred before the divergence of monocots and eudicots in angiosperm evolution. For example, *Arabidopsis thaliana* has undergone two recent WGD events, with the most recent one occurring at approximately 23 million years ago (Mya) [4]. Soybean (*Glycine max*) has also experienced two WGDs [5], which occurred at approximately 59 Mya and then 13 Mya. WGDs can duplicate entire chromosomes, thereby resulting in a large number of duplicate genes. These duplicate genes are considered to play important roles in enhancing organisms’ adaptation to the environment and promoting species diversification [6–9]. The functions of the duplicate genes have diverged remarkably throughout evolution, although most duplicate genes have been lost [10, 11].

Although many mechanisms can explain the functional divergences of the duplicate genes, the paralogous genes generated through WGDs should initially share identical sequences and chromatin environments and possess stronger expression correlations than would be found among other duplication types [12]. Theoretically, paralogs should share identical expression levels in the absence of selective pressures and stress [13], because they share identical sequences. Functional divergences and expression differences have arisen due to selective pressures and harsh environments after hundreds of millions of years of evolution [14]. The divergences in the regulatory regions of genes may have changed their expression patterns, whereas changes in the coding regions may have resulted in the acquisition of new functions [15–17]. Therefore, gene expression divergence is an important evolutionary driving force for paralogs.

Several studies have examined the relationship between the sequence and expression divergence of duplicates [17–21]. Warnefors and Kaessmann investigated the correlations between the divergence of gene and protein expression in mammals and identified several positive correlations [22]. However, a study in sunflower has indicated that there are no correlations. This study instead described decoupling between gene expression and sequence divergence [23], with similar results reported in flycatcher species [24]. Furthermore, many studies have confirmed that genes with high expression levels evolve more slowly than those with low expression levels [25], and correlations between expression divergence and selective pressure have also been reported. For example, studies in *Drosophila* indicated that positive selection is closely related to expression divergence [26], whereas others have reported that purifying selection is the primary driving force of the divergences in expression and sequence [27]. Consequently, it is important to know whether there are correlations between expression divergences of paralogs that may have resulted from selective pressure in plants.

We investigated the differential expression patterns and expression divergences of paralogs under four different types of stress (two biotics stresses and two abiotics stresses) in *Arabidopsis thaliana*. Furthermore, we identified correlations between sequence divergences and selective pressures. Lastly, we constructed co-expression networks of paralogs with different expression patterns and associated transcription factors. A workflow chart showing the different steps presented in this study can be found in Figure S1.

## Results

### Homolog identification and paralog expression classification

**Fig. 1:**
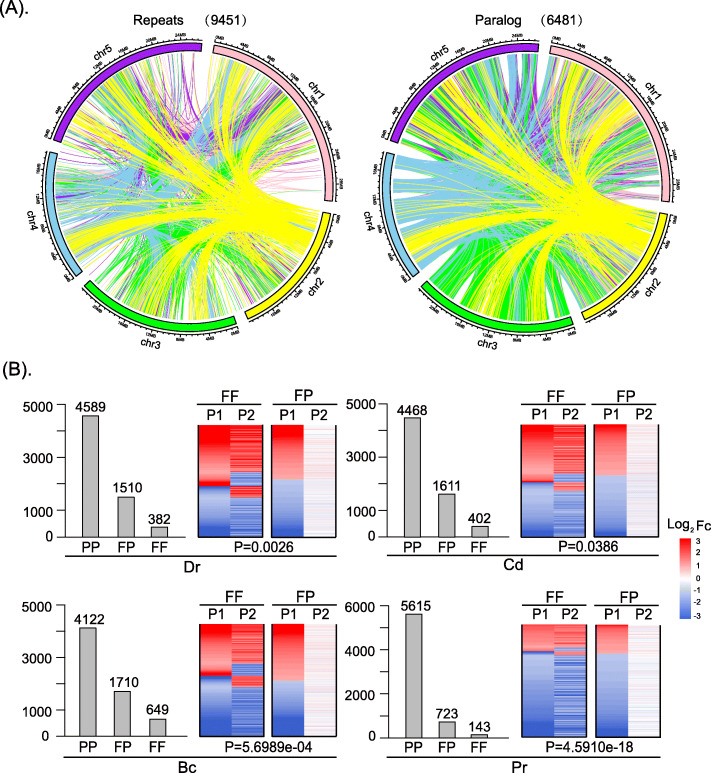
Distributions and expression classifications of the paralogs. **a** The distributions of 9451 repetitive sequences and 6481 paralogs throughout the chromosomes. **b** The identification and expression of the three types of paralogs under four different types of stress, including two biotic stresses (infection by the necrotrophic fungus *Botrytis cinerea*, *Bc* and herbivory by the chewing larvae of *Pieris rapae*, *Pr*) and two abiotic stresses (drought and cold)

We identified 6481 paralogs (paralogous gene pairs) in the model plant species *Arabidopsis thaliana* based on a homology analysis which involved 20 other species using the InParanoid 8 Software (see the Methods section for details) [28]. The list of 6481 paralogs is show in Table S1. The phylogenetic relationships of the 21 species were obtained from Lian et al. and Ren et al. [2, 29]. Thereafter, we analyzed the interactions and distributions of the paralogs and repeats in the chromosomes, respectively (Fig. 1a). The corresponding interaction information is presented in Table S2 and Table S3. The repeats of *Arabidopsis thaliana* were identified using the RepeatMasker and HashRepeatFinder tools (described in the Methods section). These results indicated that the paralogs and repeats were highly coincident with regard to their locations and interactions, and the corresponding coincidence rate was 82.4%. This which further confirmed that the paralogous gene pairs were mostly generated through genome duplications, including WGDs and small-scale duplications (SSDs) [2, 30].

Next, we classified the paralogs into three types (FF, FP or PP) (see definitions in Methods) according to their expression patterns under four different types of stress, including two biotic stresses (infection by the necrotrophic fungus *Botrytis cinerea*, *Bc*; and herbivory by the chewing larvae of *Pieris rapae*, *Pr*) and two abiotic stresses (drought [*Dr*] and cold [*Cd*]). We identified 382, 1510, and 4589 differentially expressed pairs of FF, FP, and PP paralogs in *Dr* stress; 402, 1611, and 4468 differentially expressed pairs of FF, FP, and PP paralogs in *Cd* stress; 649, 1710, and 4122 differentially expressed pairs of FF, FP, and PP paralogs in *Bc* stress; and 143, 723, and 5615 differentially expressed pairs of FF, FP, and PP paralogs in *Pr* stress, respectively (Fig. 1b). The list of FF, FP and PP paralogs under four different stresses is shown in Table S4. The statistic significances of differences in expression of FF and FP genes under the four different types of stress were examined by using Mann-Whitney *U*-test (Fig. 1b). Differences were considered significant when their *P*-value was less than 0.05. The log2|FC| values of FF and FP paralogs under four different stresses are shown in Table S5. We also investigated co-expressed FF paralogs under the four different types of stress by computing the *Pearson* coefficient *r*. The proportions of the co-expressed FF paralogs were 77.4% in *Dr* stress, 84.3% in *Cd* stress, 79% in *Bc* stress and 93% in *Pr* stress (Fig. 1b). The threshold of *Pearson* coefficient was *r >* 0.5*.*

These results showed that (1) most paralogous genes were not expressed or differentially expressed, and only a small proportion of the paralogous genes were both differentially expressed, which suggests that most paralogous genes are not involved in stress response mechanisms; and (2) the expression patterns of paralogs involved in stress response were significantly different, especially for FF and FP paralogs, which suggests that these differentially expressed paralogs (DEPs) are significantly differentially expressed in stress response; (3) most paralogs with FF expression patterns under four different types of environmental stress tend to show similar expression patterns.

### Differential expression patterns of paralogs under biotic and abiotic stress

**Fig. 2:**
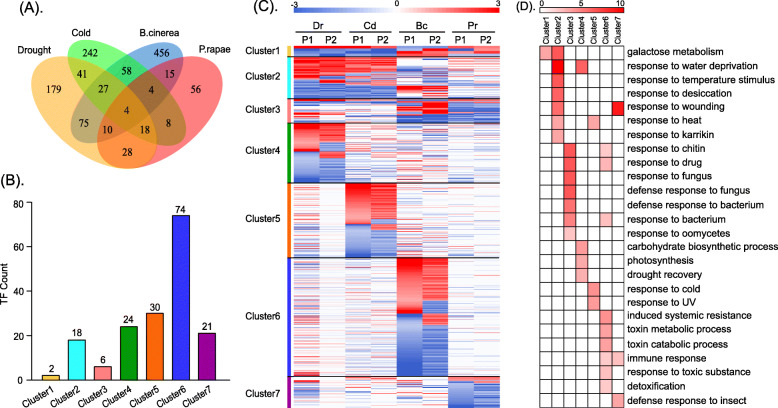
The differential expression patterns and functional enrichment of the FF paralogs under four different types of stress. **a** Venn diagram of the FF paralogs under four different types of stress. **b** The number of transcription factors in each cluster of FF paralogs. **c** Heatmap of seven expression modules of the FF paralogs under four different types of stress. Color bars represent the log2|FC| values, with red representing up-regulation and blue representing down-regulation. **d** The functional enrichment of seven clustered paralogs in the heatmap

To investigate the differential expression patterns of FF and FP paralogs under the four different types of stress, we generated a Venny diagram of their overlaps (Fig. 2 for FF, Fig. S2 for FP). We first clustered all 1052 FF paralogs and 2703 FP paralogs into seven expression modules according to their differential expression patterns under different types of stress. The log2|FC| values of seven FF and FP expression clusters are shown in Table S6 and Table S7, respectively. The corresponding heatmaps and the specific functions of the FF and FP paralogs are shownin Figs. 2 and S2, respectively. Furthermore, we identified the transcription factors (TFs) in each cluster. The FF and FP paralogs belonging to the first three clusters were differentially expressed during allfour different types of stress. The paralogs belonging to the last four clusters were differentially expressed during only one type of stress. We also performed function enrichment and KEGG analysis for the FF paralogs to assign functional categories to each module (Fig. 2d).

Cluster 1 contained four DEPs, two of which were TFs and were shared by all four types of stress (Fig. 2a-c). Functional enrichment analysis indicated that these four paralogs were mainly involved in galactose metabolism, and two of the TFs were *bHLH* transcription factors. These results indicate that plants require more energy to deal with harsh environments, which has been confirmed by a recent study [31]. Cluster 2 contained 90 differentially expressed paralogs, eighteen of which were TFs and were shared by abiotic stresses (*Dr* and *Cd*) (Fig. 2a-c). The functions of DEPs in cluster 2 were mainly involved in the response to various abiotic stresses, such as water deprivation, temperature fluctuations, and karrikin (Fig. 2d). Studies have confirmed that these genes are involved in the biosynthesis of abscisic acid, and they improved the abiotic stress tolerance in *Arabidopsis thaliana* when overexpressed [32, 33]. Karrikin, a signaling molecule, is found in smoke from burning vegetation, and it triggers seed germination for many angiosperms [34]. This may be a protective mechanism used by plants for seed development in response to harsh environmental conditions, such as drought, cold, and high salinity [35]. Cluster 3 contained 33 differentially expressed paralogs, six of which were TFs that were shared by biotic stresses (Fig. 2a-c). The corresponding functions were mainly involved in the response to various biotic stresses, such as protection from attacks by fungi, bacteria and oomycetes, as well as immunological processes. We identified five differentially expressed *WRKY* TFs (*WRKY6*, *WRKY40*, *WRKY54*, *WRKY70* and *WRKY18*), reflecting the important roles of *WRKY* TFs in the response to biotic stress. For example, *WRKY70* and *WRK54* are involved in basal defense mechanisms against *Hyaloperonospora parasitica* and disease resistance in Arabidopsis [36]. On the other hand, *WRKY6* and *WRKY40* play important roles in transducing *E-2*-hexenal perception, which is a green leaf volatile (GLV) that is produced upon wounding, herbivory or infection by pathogens [37].

With regard to clusters 4 through 7, we identified 179 (containing 24 TFs), 242 (containing 30 TFs), 456 (containing 74 TFs) and 56 paralogs (containing 21 TFs) that were differentially expressed under *Dr*, *Cd*, *Bc*, and *Pr* stress, respectively. The proportions of the co-expressed paralogs were 6.9, 6.6, 8.6 and 20.4% under *Dr*, *Cd*, *Bc* and *Pr* stress, respectively (Fig. 2a-c). The functional enrichment of cluster 4 indicated that the 179 paralogs were mainly enriched in carbohydrate biosynthesis, photosynthesis and drought recovery. Furthermore, *bHLH* negatively regulates jasmonate signaling and improves tolerance to drought stress [38]. The functions of cluster 5 were mainly enriched in the response to cold and ultraviolet light. As previously reported, these genes are involved in diurnal oscillation and beta-amylase biosynthesis, which increases the sensitivity of the PSII photochemical reaction to freezing and ambient stress in Arabidopsis [39, 40]. The functions of clusters 6 and 7 were mainly enriched in systemic resistance, toxin metabolism, immune response and protection from insects (Fig. 2d).

These results indicate that (1) paralogs with different expression clusters participate in different biological processes and have different biological functions; (2) the paralogous genes with functional redundancy were differentially expressed during the exposure to different types of stress, and (3) the expression patterns of the paralogous genes can change under different stress conditions.

### Differential expression patterns of paralogs under different degrees of the same type of stress

**Fig. 3:**
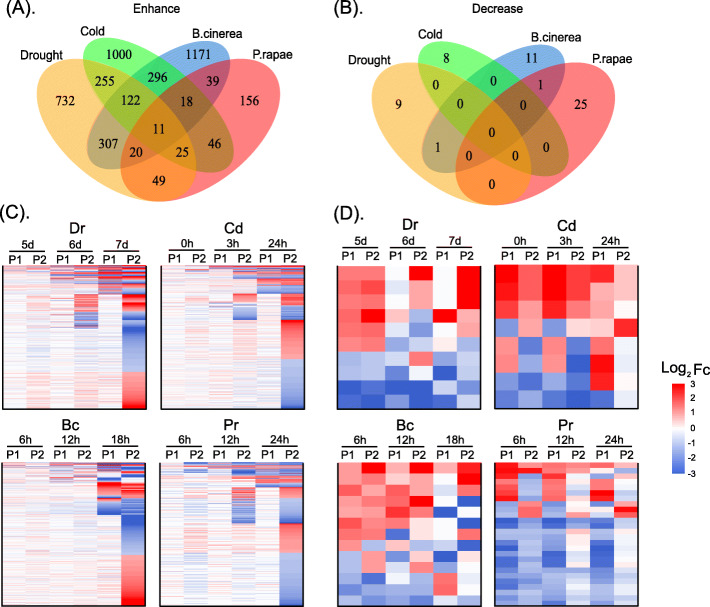
The expression pattern of enhancing and decreasing paralogs under different types of stress. **a** Venn diagram of paralogs with enhancing expression patterns under four different types of stress. **b** Venn diagram of paralogs with decreasing expression patterns under four different types of stress. **c** The heatmaps of paralogs with enhancing expression patterns under each stress condition. **d** The heatmaps of paralogs with decreasing expression patterns under each stress condition

We next investigated the effects of different degrees of stress on the expression patterns of the paralogs and classified the paralogs into two types according to expression level, which we defined as the enhancing expression pattern (PP → FP → FF) and decreasing expression pattern (FF → FP → PP) (Fig. 3). We identified 1521 and 10, 1773 and 8, 1985 and 13, and 364 and 26, enhancing and decreasing paralogs in *Dr*, *Cd*, *Bc*, and *Pr* stress, respectively (Fig. 3a, b). The log2|FC| values of paralogs with enhancing and decreasing patterns under four stresses are shown in Table S8 and Table S9.

For the enhancing expression pattern, the paralogs were not expressed or differentially expressed at the onset of different stress. With prolonged or increased stress, more paralogs became differentially expressed (Fig. 3a, c). At the strongest phase of *Dr*, *Cd*, *Bc* and *Pr* stress, the proportions of DEPs all reached 100%. The functional enrichment of the paralogs indicated that those responsive to the *Dr* stress were mainly involved in processes related to water deprivation and photosynthesis [41], those responsive to the *Cd* stress were mainly involved in processes related to temperature fluctuations and cold [42], those responsive to the *Bc* stress were mainly involved in processes related to protection from bacterial infection [43], and those responsive to the *Pr* stress were mainly involved in processes related to the defense response and immunological events [44]. Furthermore, we found that some enhancing paralogs were differentially expressed in at least two different types of stress simultaneously, and the proportions of the up-regulated paralogs in *Dr*, *Cd*, *Bc* and *Pr* co-enhanced with another type of stress were 22.1, 22.6, 14.5, and 20.1%, respectively (Fig. 3c). These results indicate that most paralogs can respond to or be activated by several types of stress. Functional enrichment analysis of the 255 paralogs that responded to both *Dr* and *Cd* stress confirmed the functional redundancy with regard to water deprivation and temperature fluctuations. The functions of the 11 paralogs (Fig. 3a) shared by the four types of stress were mainly enriched in ion homeostasis and auxin transport [45], which have been reported to be involved in a wide array of stress responses [46, 47].

For the decreasing expression pattern, the paralogs were significantly differentially expressed at the onset of different types of stress. With prolonged stress, more paralogs were not expressed or differentially expressed (Fig. 3b, d). The functional enrichment of the paralogs indicated that those responsive to *Dr* and *Cd* stress were mainly involved in processes related to monocarboxylic acid and carboxylic acid biosynthesis. Recent studies have reported that these small molecules can help plants to adapt to extreme stress conditions [48, 49].

These results indicate that the expression patterns of the paralogs vary under different types of stress as well as with different degrees of stress, suggesting that the expression levels of paralogs are not only related to the type but also the severity of stress. These results also reveal that most paralogs are differentially expressed in response to multiple stresses, suggesting that the functional redundancy of paralogs is a protective mechanism for the adaptation of plants to different stress environments throughout evolution.

### Co-expression networks of DE paralogs and transcriptional factors under different types of stress

**Fig. 4:**
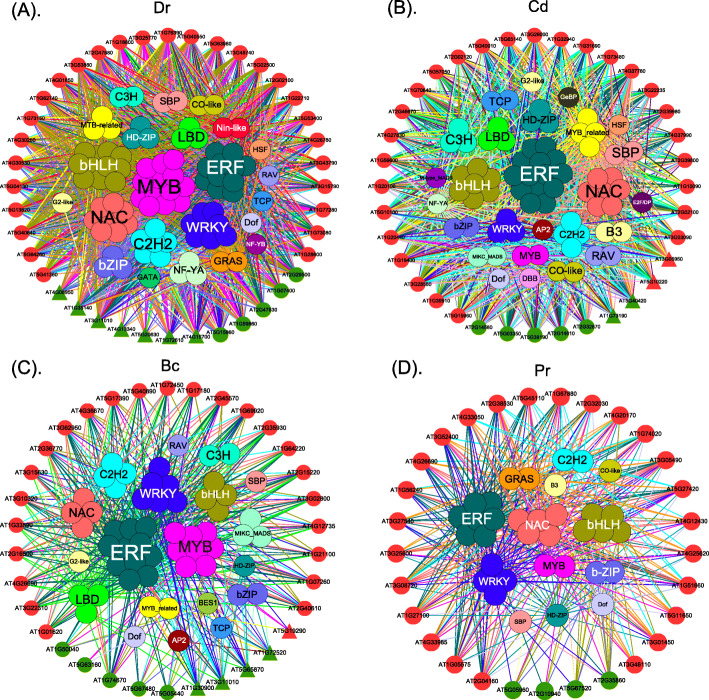
The co-expression networks of DEPs with enhancing and decreasing patterns under four different types of stress. The outer circle represents differentially expressed paralogs. Red and green represent paralogs with enhancing and decreasing expression patterns, respectively. Triangles represent the up-regulated genes, while circles represent the down-regulated genes. The inner circle represents the co-expressed TFs

To understand how transcription factors (TFs) regulate the expressed of DEPs in response to stress, we constructed co-expression networks for *Dr*, *Cd*, *Bc* and *Pr* stresses (Fig. 4).

The co-expression networks revealed several important insights. Firstly, among the enhancing and decreasing expression patterns of down-regulated DEPs under *Dr*, *Cd*, *Bc* and *Pr* stress, DEPs with both enhancing and decreasing patterns showed low expression, except for DEPs with a decreasing pattern under *Dr* stress. Secondly, the top three TFs co-expressed with DEPs were *MYB*, *ERF* and *bHLH* under *Dr* stress (Fig. 4a); *ERF*, *bHLH* and *NAC* under *Cd* stress (Fig. 4b); *ERF*, *MYB* and *WRKY* under *Bc* stress (Fig. 4c); and *ERF*, *NAC* and *MYB* under *Pr* stress (Fig. 4d). Previous studies have reported that *ERF* plays important roles in responses to both biotic and abiotic stresses [50–52]. For example, *ERF9* protects Arabidopsis from necrotrophic fungi, and post-anaerobic reoxygenation—the main defense mechanism in plants [53]—is regulated by *ERF96* [54]. A study has also confirmed that *bHLH* can mediate the trade-off between abiotic and biotic molecular pattern-triggered immunity in Arabidopsis [55, 56]. However, *MYB* is mainly involved in response to biotic stress [57, 58]. Thirdly, we identified specific TFs under different types of stress. For example, *NIN-LIKE* is a master regulator of the response of Arabidopsis to *Dr* stress [59]. *E2FD/DEL2* controls cell proliferation in Arabidopsis during exposure to *Cd* stress [60]. *BES1* promotes brassinosteroid signaling and development in *Arabidopsis thaliana* during exposure to *Bc* stress [61]. Finally, there were more interactions between DEPs and TFs with an enhancing expression pattern than those with a decreasing expression pattern (Fig. 4). The increased number of interactions indicated that more TFs regulated the responses of the paralogs to the enhancing severity of stress. These results are very helpful for understanding the regulatory mechanisms of TFs with regard to the responses of paralogs to stress.

### Expression divergences positively correlate with sequence divergences

**Fig. 5:**
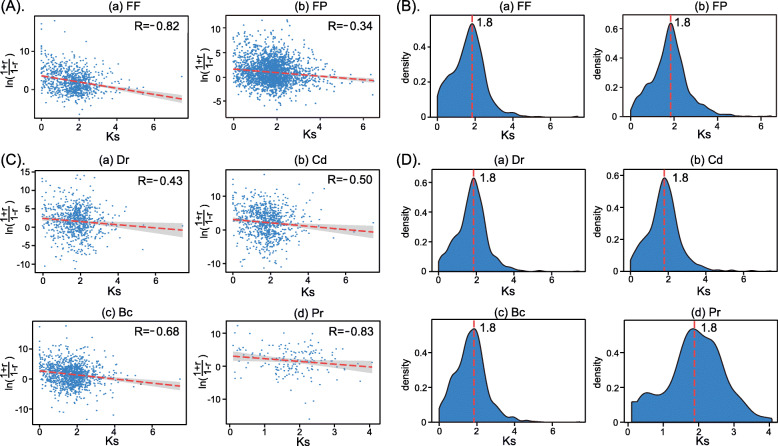
The regression results of the expression divergences and sequence divergences. (A) The regression results of FF (a) and FP (b) paralogs under all four types of stress. (B) The density plot of *Ks* values of FF (a) and FP (b) paralogs under all four types of stress. (C) The regression results of paralogs with enhancing and decreasing expression under each stress condition. (a) Dr., (b) Cd, (c) Bc and (d) Pr. (D) The density plot of *Ks* values of paralogs with enhancing and decreasing expression under each of the four types of stress. (a) Dr., (b) Cd, (c) Bc and (d) Pr

We continued our study by investigating whether there were positive or negative correlations between expression divergences and sequence divergences [62]. First, the paralogs with FF and FP expression patterns were investigated. To estimate the sequence divergence between paralogs, we computed the synonymous (*Ks*) substitution rate, which is recognized as a proxy of the sequence divergence time. According to previous studies [21, 62], we used the rescaled Pearson’s correlation coefficient *r*’ to perform linear regression analysis (see the Methods section for details). The regression results of the expression levels of FF and FP paralogs and the *Ks* rates are shown in Fig. 5.

We found a significant negative correlation between the rescaled *r*’ and *Ks* values for FF and FP gene pairs (*P* < 0.001, *U*-test, Fig. 5A). The negative correlation between the *r*’ and *Ks* values was indicative of a positive correlation between expression divergence and sequence divergence. These results indicate that the expression divergences of both FF and FP gene pairs were positively correlated with sequence divergences. Furthermore, we investigated the distribution of *Ks* values for FF and FP paralogs and identified one peak with a value of 1.8 in the density plot (Fig. 5B). These results indicate that the gene pairs originating at a value of 1.8 experienced a large amount of synonymous substitution. More than 80% of FF and FP paralogs had *Ks* values larger than 1.0, suggesting that they have persisted for a relatively long evolutionary duration time and are highly divergent. In addition, the gene pairs near the *Ks* peak probably experienced larger expression divergences [63].

We also investigated the correlations of DEPs with enhancing and decreasing expression patterns under *Dr, Cd, Bc* and *Pr* stress. We identified a negative correlation between the expression divergences and *Ks* value for all four types of stress (*P* < 0.001, *U*-test, Fig. 5C). These results indicate that the expression divergences of DEPs in response to stress were positively correlated with sequence divergences. Furthermore, a density plot of the corresponding *Ka* and *Ks* values had a *Ks* peak value of 1.8 (Fig. 5D), indicating that these genes have persisted for a relatively long evolutionary duration and are highly divergent.

In summary, this study reveals new correlations between the expression divergences and sequence divergences of paralogous genes, which adds to the current understanding of the evolutionary mechanisms behind stress adaptation in plants.

### Selective pressures are correlated with the expression divergences of paralogs

**Fig. 6:**
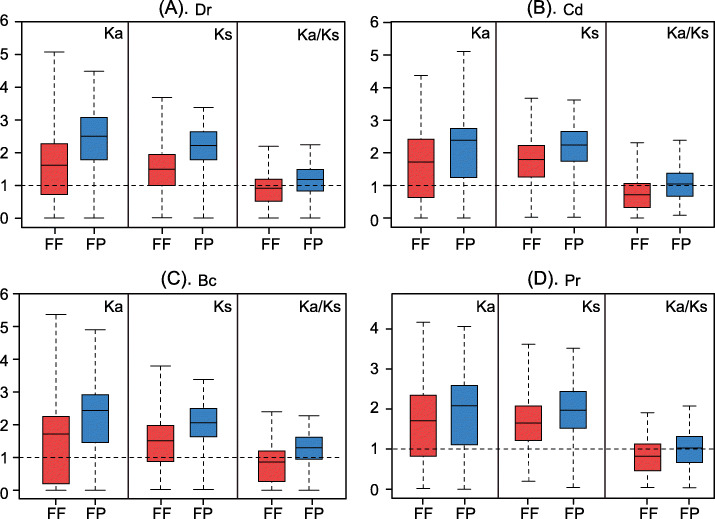
Boxplot of *Ka, Ks* and *Ka/Ks* of FF and FP DEPs under four different stress conditions

We next investigated whether there were correlations between expression divergences and selective pressures of the paralogous genes. To infer selective pressures, we used FF and FP DEPs under *Dr*, *Cd*, *Bc* and *Pr* stress to compute their non-synonymous/synonymous substitutions rate ratios (*Ka*/*Ks*). The boxplot of *Ka* and *Ks* values, as well as the *Ka/Ks* ratios, of FF and FP DEPs under the four types of stress is shown in Fig. 6.

These results revealed two important insights. First, the median value of the *Ka/Ks* ratio for FP was consistently larger than 1.0, but that of FF was smaller than 1.0 for all four types of stress, indicating that the FP gene pairs underwent positive selection but the FF gene pairs underwent purifying/negative selection. Secondly, the *Ka* and *Ks* values of FP for all four types of stress were consistently larger than those of FF, revealing that the FP gene pairs experienced more non-synonymous/synonymous substitutions and were evolutionarily older than the FF gene pairs. To ensure that the phenomena we observed were not due to chance, we compared our results with a randomized experiment containing an equal number of randomized gene pairs (Fig. S3, Methods), and found that the *Ka/Ks* ratio of FF was consistently smaller than 1.0 and that of the randomized experiment [29], but the *Ka/Ks* ratio of FP was consistently larger than 1.0 and that of the randomized experiment (*P*< 10^−4^). Statistical significance was determined by 10,000 randomized comparisons.

These results indicate that FF paralogous pairs experienced relaxed selection constraints and retained functional redundancy, but FP paralogous pairs experienced strong positive selection and more sequence divergence, which led to functional divergence. These findings suggest that paralogs with different expression patterns likely experienced different selection constraints.

## Discussion

### Sequence divergences of the paralogs support the phylogenetic relationships among species

**Fig. 7:**
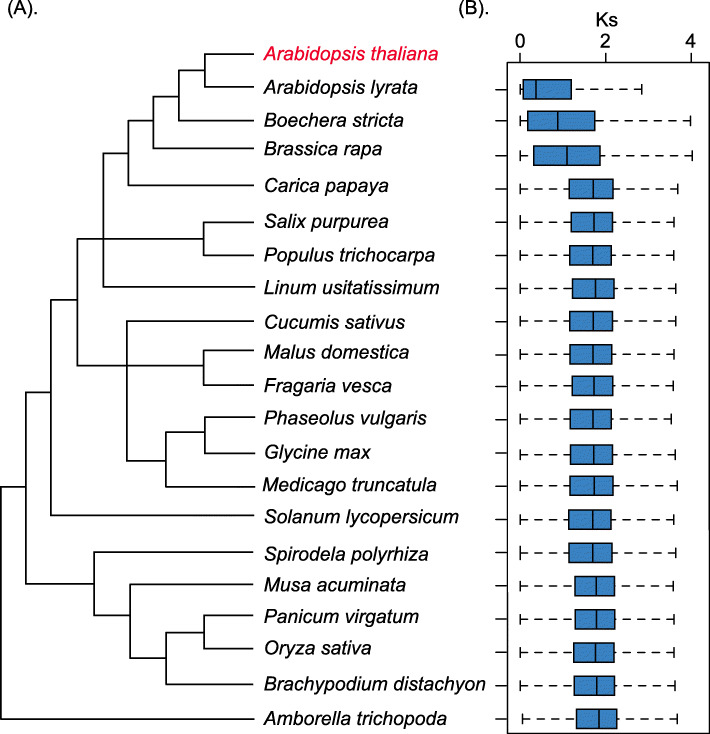
**a** The phylogenetic tree of the 21 species presented in this study. **b** The boxplot of the *Ks* values of the paralogs between *Arabidopsis thaliana* and the other 20 species

To investigate the correlations between sequence divergences and phylogenetic relationships, we examined the synonymous substitution rate (*Ks*) of paralogs between *Arabidopsis thaliana* and 20 other species (Fig. 7a). The corresponding boxplot of *Ks* values is shown in Fig. 7b. Generally, smaller *Ks* values indicated less synonymous substitutions and divergences as well as stronger phylogenetic relationships. The results in Fig. 7 show that three species, *Arabidopsis lyrata*, *Boechera stricta*, and *Brassica rapa*, had much smaller *Ks* values (0.3707, 0.878, and 0.905, respectively) for *Arabidopsis thaliana*, as compared with 17 other species (all larger than 1.0). This indicates that the genomes of these three species display less divergence and closer phylogenetic relationships with *Arabidopsis thaliana*, which is consistent with the phylogenetic results of angiosperms [64]. Furthermore, we identified an inversely proportional correlation between species conservation and family size (Fig. S4). The family size of the paralogs significantly decreased as the occurrence of the species increased. A recent study has proposed a model of exponential decrease of duplicate genes over time [2]. Further studies are needed to investigate whether the relationship between species conservation and family size of the paralogs fits the exponential decay model, as these results may improve our understanding of the evolution of the duplicate genes.

### Conserved domains and cis-elements

A recent report has confirmed that the expression divergence of the duplicated genes is primarily attributed to alterations in cis-elements [65], which have been proposed to mediate the expression divergence of genes in rice [66]. To further assess the impacts of cis-elements on expression divergence, we investigated the conserved domains and cis-elements of the paralogs in all 21 species.

**Fig. 8:**
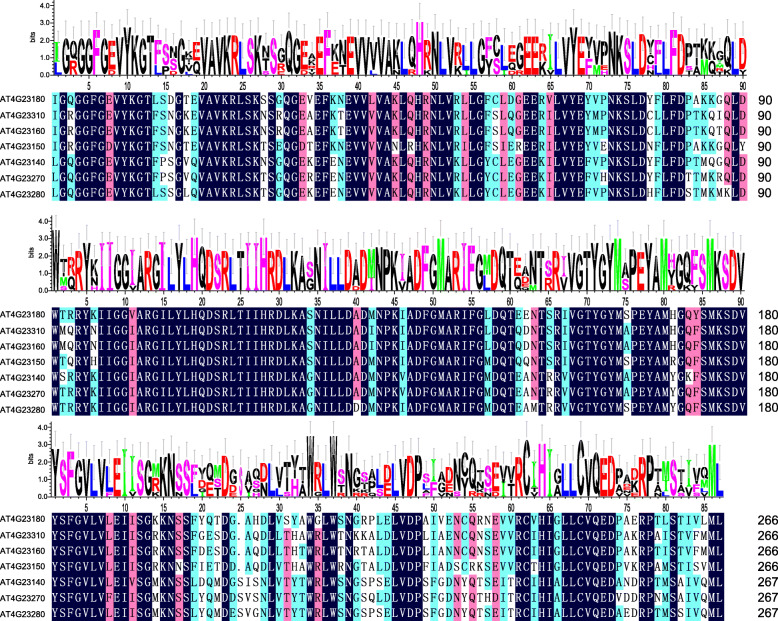
The conserved protein domain sequences of the paralogs in all 21 species

**Fig. 9:**
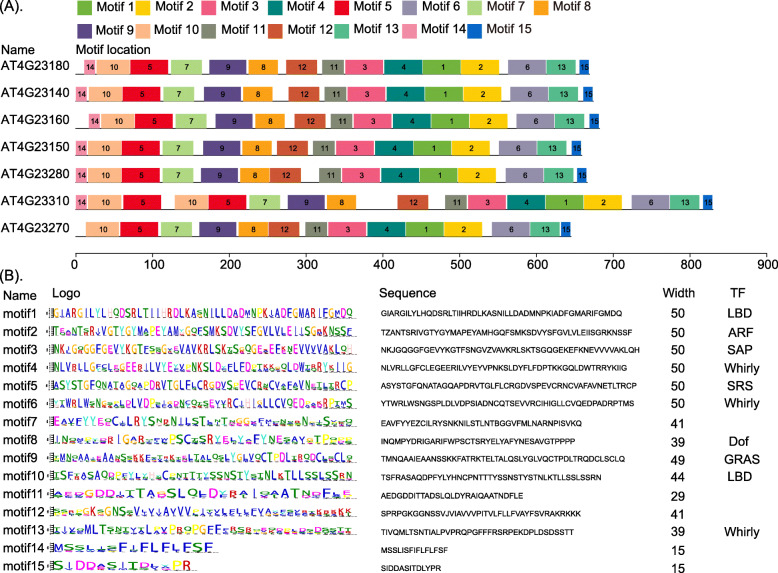
The conserved motifs of the paralogs in all 21 species

We identified one paralogous gene family with seven genes in all 21 species and used the CDD Database to identify their conserved domains. The most highly conserved protein domains were the catalytic domain of the serine/threonine kinases (STKs), interleukin-1 receptor associated kinases and related STKs (STKc-IRAK) (Fig. 8). The STKs catalyze the transfer of the gamma-phosphoryl group from ATP to serine/threonine residues on the protein substrates. IRAKs are involved in the Toll-like receptor (TLR) and interleukin-1 (IL-1) signaling pathways. Thus, they regulate innate immune responses and inflammation [67, 68]. Using the MEME software, we identified 15 conserved motifs of STKc-IRAK, and found that most motifs were widespread in TFs, such as *LBD*, *ARF*, *SAP*, *Whirly*, *SRS*, *Dof* and *GRAS* (Fig. 9a, b). Furthermore, the seven genes in all 21 species shared similar motif structures and gene lengths.

**Fig. 10:**
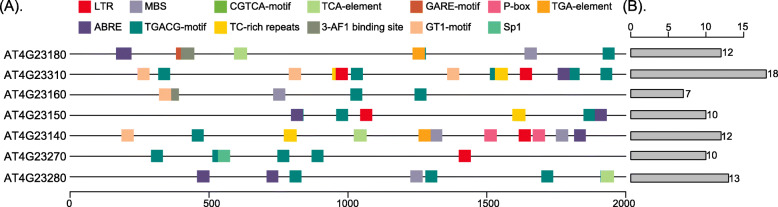
**a** The top ten cis-elements of STKc_IRAK in the 2000-bp promoter sequence. **b** The number of cis-elements in each gene

We used PlantCARE to predict cis-element variations of the STKc-IRAK gene family and identified 13 cis-elements related to stress in the 2000-bp promoter sequence of the paralogous gene family (Fig. 10). The top ten components are shown in Fig. 10a, and they include a low temperature response component (*LTR*), *MYB* binding site involved in the drought induction (*MBS*), MeJA reaction component (*CGTCA-motif*), salicylic acid reaction component (*TCA-element*), gibberellin reaction component (*GARE-motif* and *P-box*), auxin response element (*TGA-element*), abscisic acid reaction component (*ABRE*), MeJA element (*TGACG-motif*), stress response element (*TC-rich repeats*) and optical response elements (*3-AF1 binding site, GT1-motif*, and *Sp1*). The number of cis-elements identified in each gene is shown in Fig. 10b. Among them, the top two elements were the *CGTCA-motif* and *TGACG-motif*, accounting for 25% for all elements. These cis-elements are all related to stress, which suggests that they may be involved in the transcriptional control of abiotic stresses and hormonal responses [69].

## Conclusion

In this study, we analyzed the expression patterns of paralogous genes under different types of stress and investigated the correlations between the divergences in expression and sequence of the paralogs. Firstly, we analyzed the differential expression patterns of the paralogs under four different stresses (*Dr, Cd, Bc* and *Pr*) and classified them into three types according to their expression patterns. Secondly, we analyzed their differential expression patterns under different degrees of stress and constructed corresponding co-expression networks of differentially expressed paralogs and TFs. Thirdly, we investigated the correlations between the divergences in expression and sequence and identified positive correlations between the expression divergences and sequence divergences. Lastly, we found that paralogs with different expression patterns likely experienced different selection constraints. FF paralogous pairs likely experienced relaxed selection constraints, while FP paralogous pairs experienced strong positive selection. These results suggest that paralogs which experienced relaxed selection tend to be functionally redundant while those which experienced strong positive selection tended to show more sequence divergence, Overall, these results provide new insightsinto the differential expression patterns of paralogs in response to environmental stresses and how those expression patterns relate to sequence divergences.

## Methods

### Homolog identification and paralog classification

We used the homolog analysis software InParanoid 8 with default parameters to identify paralogous gene pairs between *Arabidopsis thaliana* and 20 other species according to their phylogenetic relationships (Fig. 7a) [28]. The genomes and annotation files of Arabidopsis and the 20 other species were all downloaded from the EnsemblPlant (http://plants.ensembl.org) and UniProt (https://www.uniprot.org/) database. For detailed version information, For detailed version information, please refer to the attached Table S10. Among the 20 orthologs homology comparison results, multiple Arabidopsis genes corresponding to one ortholog gene were screened as candidate paralogs. In order to analyze the expression differences between a pair of genes, we selected the first two paralogs gene pairs with the highest similarity in each family as a preliminary identification and removed redundant duplicates. The originally identified homolog pairs were verified by BLAST alignment using the full-length amino acid. According to the e-value and similarity, homologous gene pairs with e-value<10e-5 and similarity ≥50% were selected. The screening results were further verified with paralogs in the EnsemblPlant databases (Table S1). After removing the identical gene pairs, 6481 paralogous gene pairs (paralogs) remained. Thereafter, we classified each paralogous gene pair into one of three types (FF, FP or PP) according to whether it was differentially expressed under different stress conditions. FF paralogs refer to paralogous gene pairs in which both genes in a pair were differentially expressed. FP paralogs refer to paralogous gene pairs in which one gene in a pair was differentially expressed and the other was not expressed or differentially expressed. PP paralogs refer to paralogous gene pairs in which both genes in a pair were not expressed or differentially expressed.

### Transcriptome analysis

The transcriptome data of *Arabidopsis thaliana* under drought stress, cold stress, infection by the necrotrophic fungus *Botrytis cinerea,* and herbivory by the chewing larvae of *Pieris rapae* were obtained from the Chinese Academy of Sciences with Bio-Project Accession No. PRJNA525452 (https://www.ncbi.nlm.nih.gov/bioproject/525452) [70]. Three time points were selected for each stress condition, with a separate control for each. See Table S11 for transcriptome data. At each time point, the transcriptional response to each single and sequential stress was compared with an untreated control or a mock-treated control. We first used Trimmomatic-0.36 software to remove the low-quality RNA-sequencing reads, and then used HISAT (Hierarchical Indexing for Spliced Alignment of Transcripts) 2–2.0.4 to map clean reads to reference genomes with default parameters for bam file generation. The expression levels of all mapped reads were normalized by FPKM (Fragments Per Kilobase of transcript per Million mapped reads) methods. Cufflinks (V2.2.0) software was then used to generate FPKM values for each gene. EdgeR was used to identify differentially expressed genes (DEGs) under four different types of stress with parameters padj< 0.05 and |log2FC| > 1 [71]. For determining the maximum dynamic range of stress response, the response to each of the four stresses was monitored in a different time frame of three time points, depending on how quickly the stress response developed. At each time point, the transcriptional response to each single and sequential stress was compared with an untreated control (for treatments not involving *B. cinerea*) or a mock-treated control (100% relative humidity conditions, as was uesd in *B. cinerea* treatments) for comparison. Control plants were sampled at the same time as stress-treated plants [70]. For differential expression pattern, we used transcriptome data at 7_d for *Dr* stress, 24_h for *Cd* stress, 18_h for *Bc* stress and 24_h for *Pr* stress. For enhancing and decreasing expression pattern analysis, we used transcriptome data at 5_d, 6_d and 7_d for *Dr* stress, 0_h, 3_h and 24_h for *Cd* stress, 6_h, 12_h and 18_h for *Bc* stress, and 6_h, 12_h and 24_h for *Pr* stress.

### Interactions and distribution analysis

We used the RepeatMasker and HashRepeatFinder tools to identify repetitive sequences in *Arabidopsis thaliana*. The threshold of similar repetitive sequences was set to 85%, and repeats shorter than 150 nucleotides were removed. We determined the locations of the repeats and paralogs on the chromosomes using annotation data and used the R packages GlobalOptions and Circlize to identify interactions and distributions on the chromosomes.

### Weighted gene co-expression network analysis

The weighted gene co-expression network analysis (WGCNA) package within R summarizes and standardizes the methods and functions for co-expression network analysis [72]. The WGCNA network construction tool was used to generate the nodes and edges of the genes by computing the correlations of the expression values. The nodes corresponded to genes, and the edges were determined by pairwise correlations between gene expression levels. The corresponding calling function within the R package was ‘blockwiseModules’. The parameters were set as follows: powers = 10, minModuleSize = 30 and mergeCutHeight = 0.25. Other parameters were kept at their default settings. The nodes with a correlation of *r* < 0.5 and edges with a weighted threshold of < 0.3 were removed. Afterwards, the Cytoscape tool (https://cytoscape.org/) was used to plot the interactions using the nodes and edges of conserved genes.

### Expression and sequence divergence analysis

The non-synonymous (*Ka*) and synonymous (*Ks*) substitutions of each paralog were computed using the ‘*dnds*’ function within MATLAB. *Ka/Ks* > 1 indicates that the gene experienced positive selection, *Ka/Ks* < 1 indicates that the gene experienced negative selection, and *Ka/Ks* = 1 indicates that the gene experienced selection [73]. The boxplots of *Ka* and *Ks* values were generated using the ‘*ggplot2*’ function within R. The Pearson coefficient *r* of the expression level of each paralogous gene pair was computed using the ‘*corr*’ function within MATLAB using the following equation:

$$ r=\frac{\sum XY-\frac{1}{N}\sum Y\sum Y}{\sqrt{\left(\sum {X}^2-\frac{1}{N}{\left(\sum X\right)}^2\right)\left(\sum {Y}^2-\frac{1}{N}{\left(\sum Y\right)}^2\right)}} $$

where *X* and *Y* represent the expression data of the two genes at different time points.

Expression divergence was measured using the rescaled Pearson coefficient *r*^′^ [36, 62], which is more appropriate for linear regression analysis.

$$ {r}^{\prime }=\frac{\ln \left(1+r\right)}{1-r} $$

Linear regression analysis was performed using the ‘*lm*’ function within R, with the rescaled *r*′. The negative regression coefficient between *r*′ and *Ks* (or *Ka*) represents a positive relationship between expression level and *Ks* (or *Ka*) value.

### Randomized experiments

We simulated randomized experiments to test the statistical significance of *Ka* and *Ks* for the FF and FP paralogs [29]. When the selective pressure was not characteristic of the FF or FP gene pairs, the results of the randomized experiment and real data were similar. To achieve this, we randomly generated an equal number of FF and FP gene pairs for each stress condition from 6481 paralogs. We repeated the randomized experiment 10,000 times to evaluate the intrachromosomal colocalization of these random pairs. For example, to test the significance of the *Ks* value for 382 FF paralogs under *Dr* stress, we randomly generated 382 gene pairs from the 6481 paralogs, and computed their *Ks* values, with 10,000 replications. The frequency distributions of the *Ka* and *Ks* rates, as well as the *Ka/Ks* ratio, with 0.1 steps are shown in Fig. S3.

### Statistical methods

The Mann-Whitney *U*-test (function ‘ranksum’ in software‘MATLAB’ version R2016b) was used to examine the statistical significance between two samples, with a default significance level of 0.05. The Mann-Whitney U-test is a nonparametric test for equality of population medians of two independent samples. The main advantage of this test is that it makes no assumption that the samples are from normal distributions.

### Cis-element and conserved domain analysis

The online platform PlantCARE (http://bioinformatics.psb.ugent.be/webtools/plantcare/html) was used for cis-element analysis utilizing the 2000 bp promoter regions of the seven paralogs [74]. The Multiple Em for Motif Elicitation (MEME) software (http://meme-suite.org/tools/meme) was used for motif discovery. The motif number was 15, and the motif width was 50 amino acids. The Conserved Domain Database (CCD, https://www.ncbi.nlm.nih.gov/cdd/) was used to analyze the conserved domain sequences [75]. Functional enrichment was performed by using Metascape tools [76], and the resulting *P* values were adjusted to Q values by the Benjamini–Hochberg correction with a false discovery rate of 5%.

### Availability of data and materials

The genetic data of the 21 species are listed in Fig. 7a, including the CDS sequences and annotation data, which were downloaded from the EnsemblPlants (http://plants.ensembl.org/) and UniProt (https://www.uniprot.org/) database. In addition, 2296 transcription factors (1717 loci) of *Arabidopsis thaliana* were downloaded from the Plant Transcription Factor Database (http://planttfdb.cbi.pku.edu.cn/index.php).

## Supplementary information

**Additional file 1: Table S1.** The gene list of 6,481 paralogs.

**Additional file 2: Table S2.** The interaction information of paralogs.

**Additional file 3: Table S3.** The interaction information of repeats.

**Additional file 4: Table S4.** The list of FF, FP and PP paralogs under four different stresses.

**Additional file 5: Table S5.** The log2FC values of FF and FP paralogs under four different stresses.

**Additional file 6: Table S6.** The log2FC values of seven FF paralogs expression clusters.

**Additional file 7: Table S7.** The log2FC values of seven FP paralogs expression clusters.

**Additional file 8: Table S8.** The log2FC values of paralogs in enhancing patterns under four stresses.

**Additional file 9: Table S9.** The log2FC values of paralogs in decreasing patterns under four stresses.

**Additional file 10: Table S10.** Version information for 21 species.

**Additional file 11: Table S11.** The transcriptome information of four stresses.

**Additional file 12: Figure S1.** Workflow chart showing the different steps undertaken in this study.

**Additional file 13: Figure S2.** The differential expression patterns of the FP paralogs under four different types of stress. (A). A Venn diagram of the FP paralogs under four different types of stress. (B). The number of transcription factors in each cluster of FP paralogs. (C). A heatmap of seven expression modules of the FP paralogs under four different types of stress.

**Additional file 14: Figure S3.** The frequency distributions of 10,000 repetitions of the randomized experiment for determining the *Ka* and *Ks* values, as well as the *Ka/Ks* ratio of FF and FP DEPs under four different types of stress. Navy blue corresponds to the randomized experiments, whereas the red dashed line corresponds to the real values.

**Additional file 15: Figure S4.** The inversely proportional correlations between species conservation and family size of the paralogous gene pairs.

## Abbreviations

WGD: Whole genome duplication
SSDs: Small-scale duplications
FF: FF paralogs refer to paralogous gene pairs in which both genes in a pair were differentially expressed
FP: FP paralogs refer to paralogous gene pairs in which one gene in a pair was differentially expressed and the other was not expressed or differentially expressed
PP: PP paralogs refer to paralogous gene pairs in which both genes in a pair were not expressed or differentially expressed
*Dr*: *drought*
*Cd*: *cold*
*Bc*: *botrytis cinerea*
*Pr*: *Pieris rapae*
DEP: Differentially expressed paralog
KEGG: Kyoto encyclopedia of genes and genomes
TF: Transcription factor
Ks: Synonymous
Ka: Non-synonymous
DEG: Differentially expressed gene
FC: Fold change
WGCNA: Weighted gene co-expression network analysis
