# Supplementary figures and images for: The differential expression patterns of paralogs in response to stresses indicate expression and sequence divergences
Source: BMC Plant Biol. 2020 Jun 16;20:277. doi: 10.1186/s12870-020-02460-x (PMC7298774; doi:10.1186/s12870-020-02460-x)

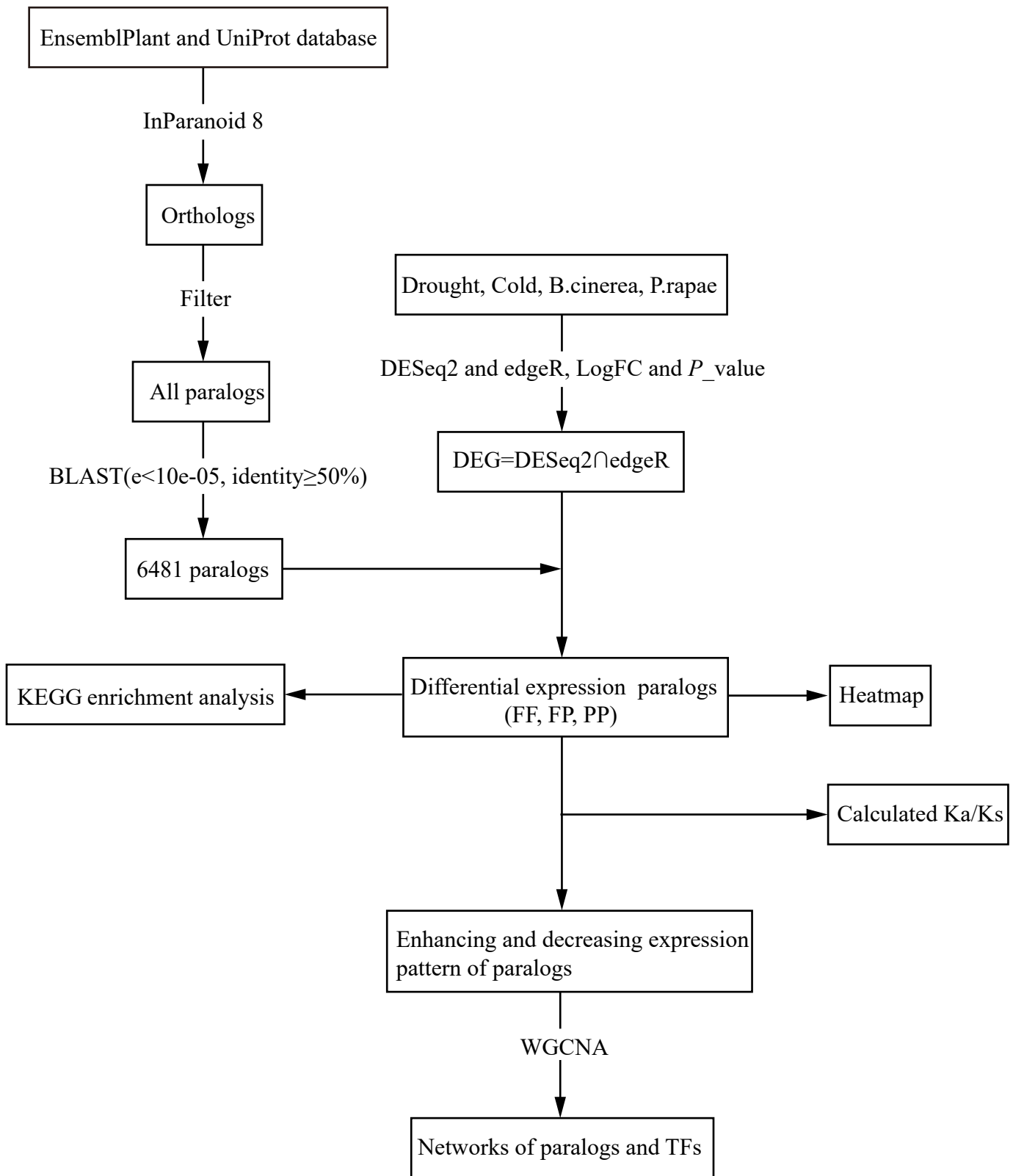

Supplement: Supplementary file 12 — Additional file 12: Figure S1. Workflow chart showing the different steps undertaken in this study. [file 12870_2020_2460_MOESM12_ESM.pdf]

(A).

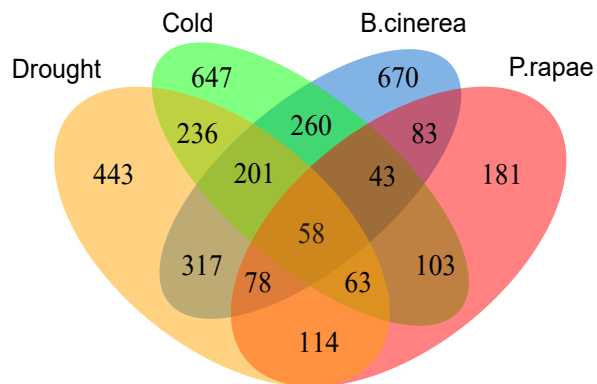

(B).

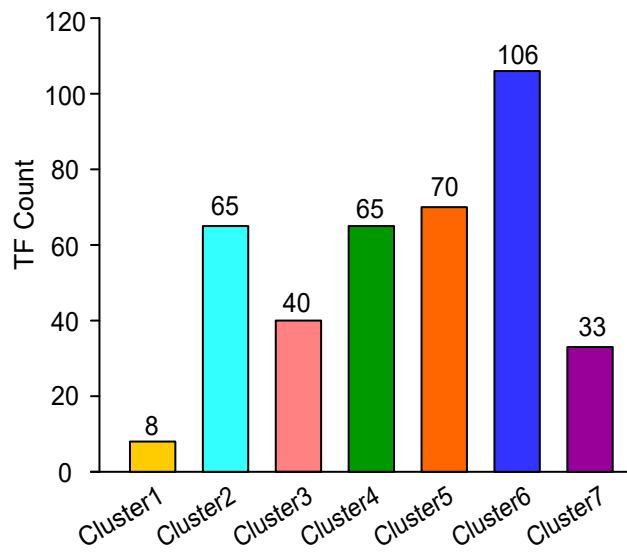

(C).

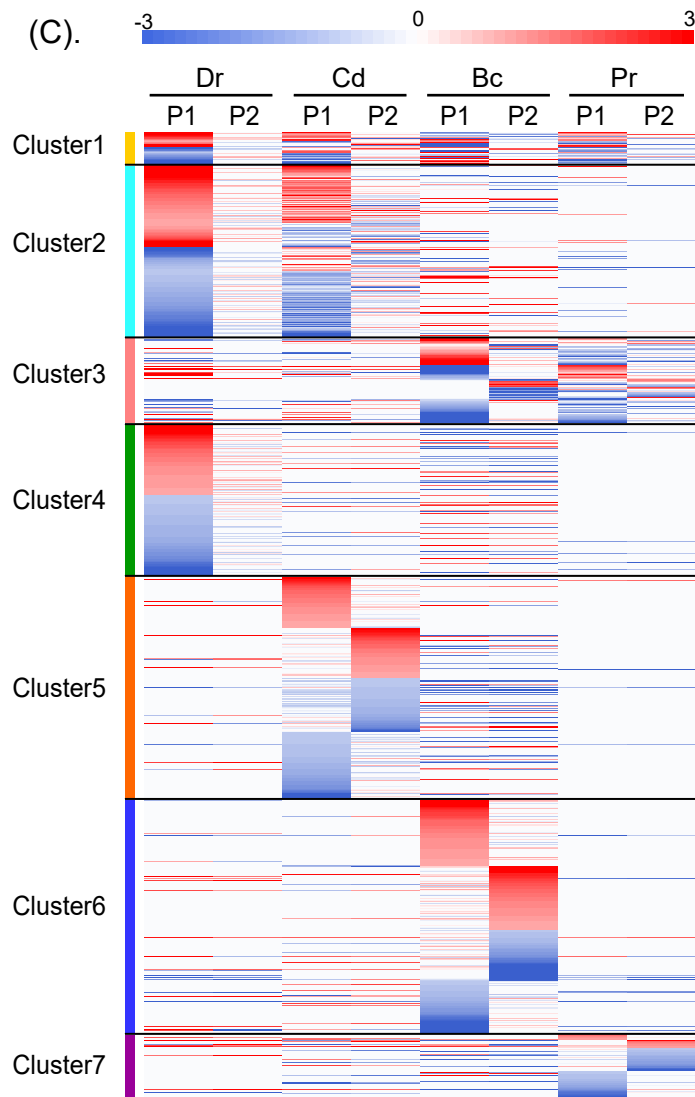

Supplement: Supplementary file 13 — Additional file 13: Figure S2. The differential expression patterns of the FP paralogs under four different types of stress. (A). A Venn diagram of the FP paralogs under four different types of stress. (B). The number of transcription factors in each cluster of FP paralogs. (C). A heatmap of seven expression modules of the FP paralogs under four different types of stress. [file 12870_2020_2460_MOESM13_ESM.pdf]

(A). Dr

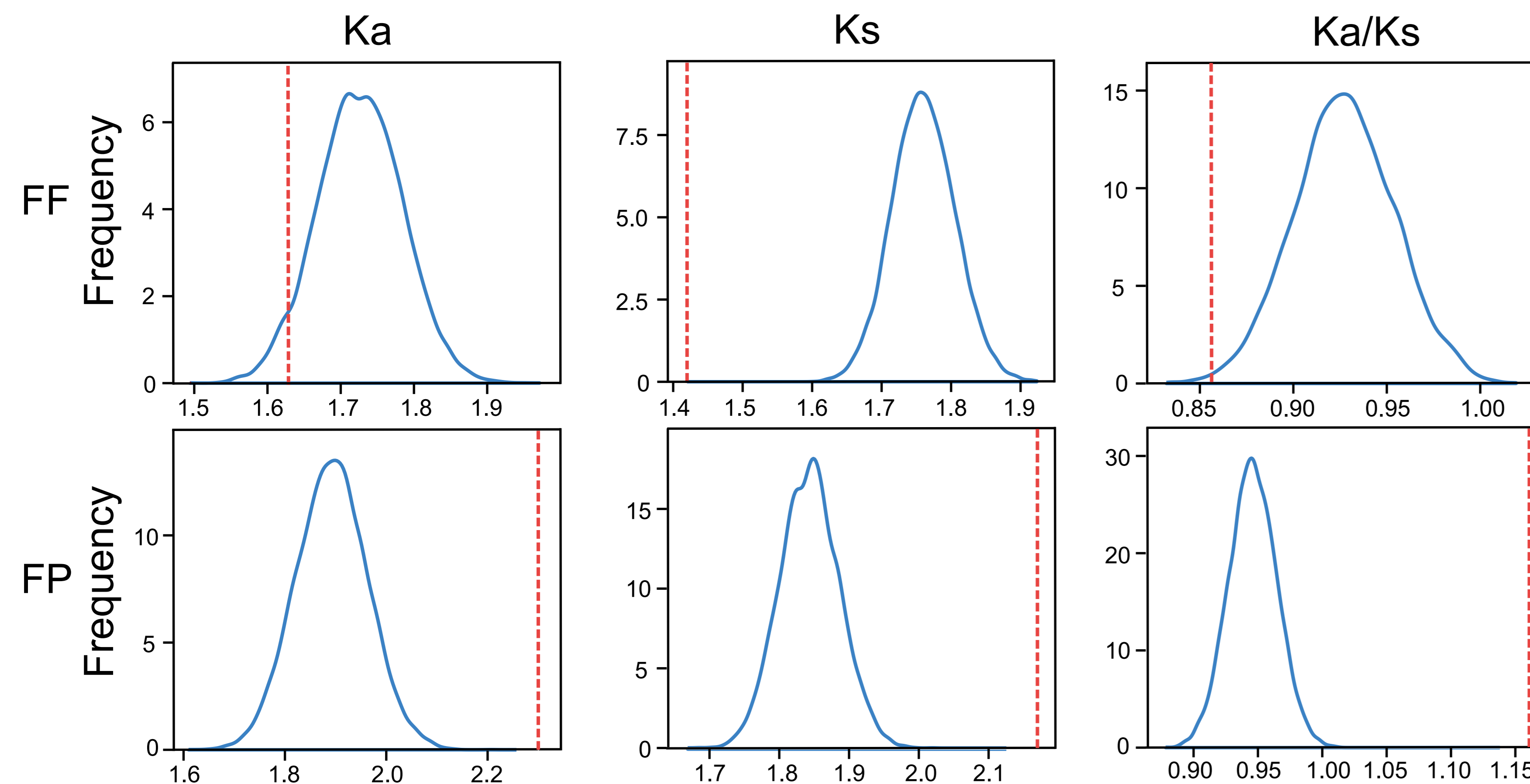

(B). Cd

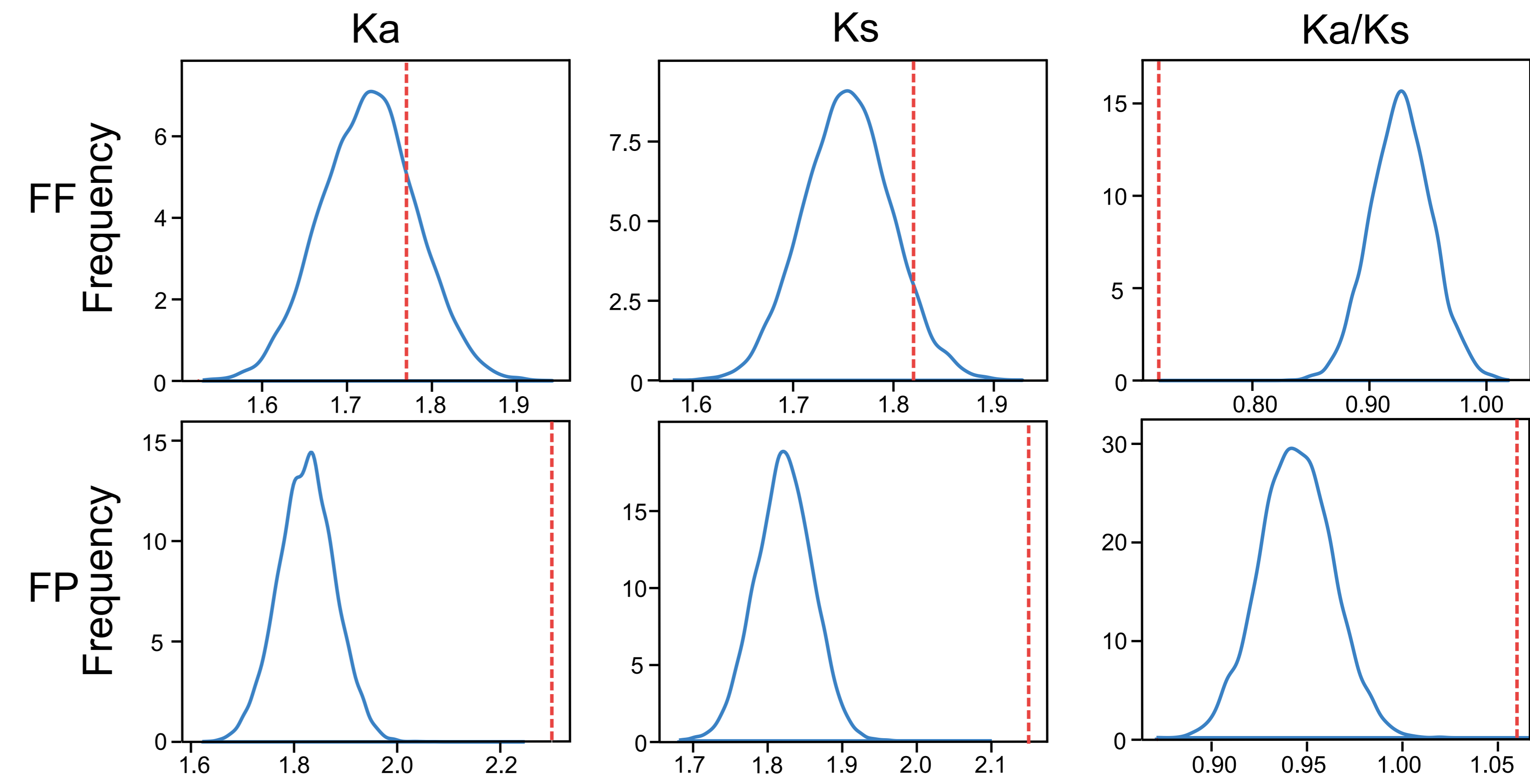

(C). Bc

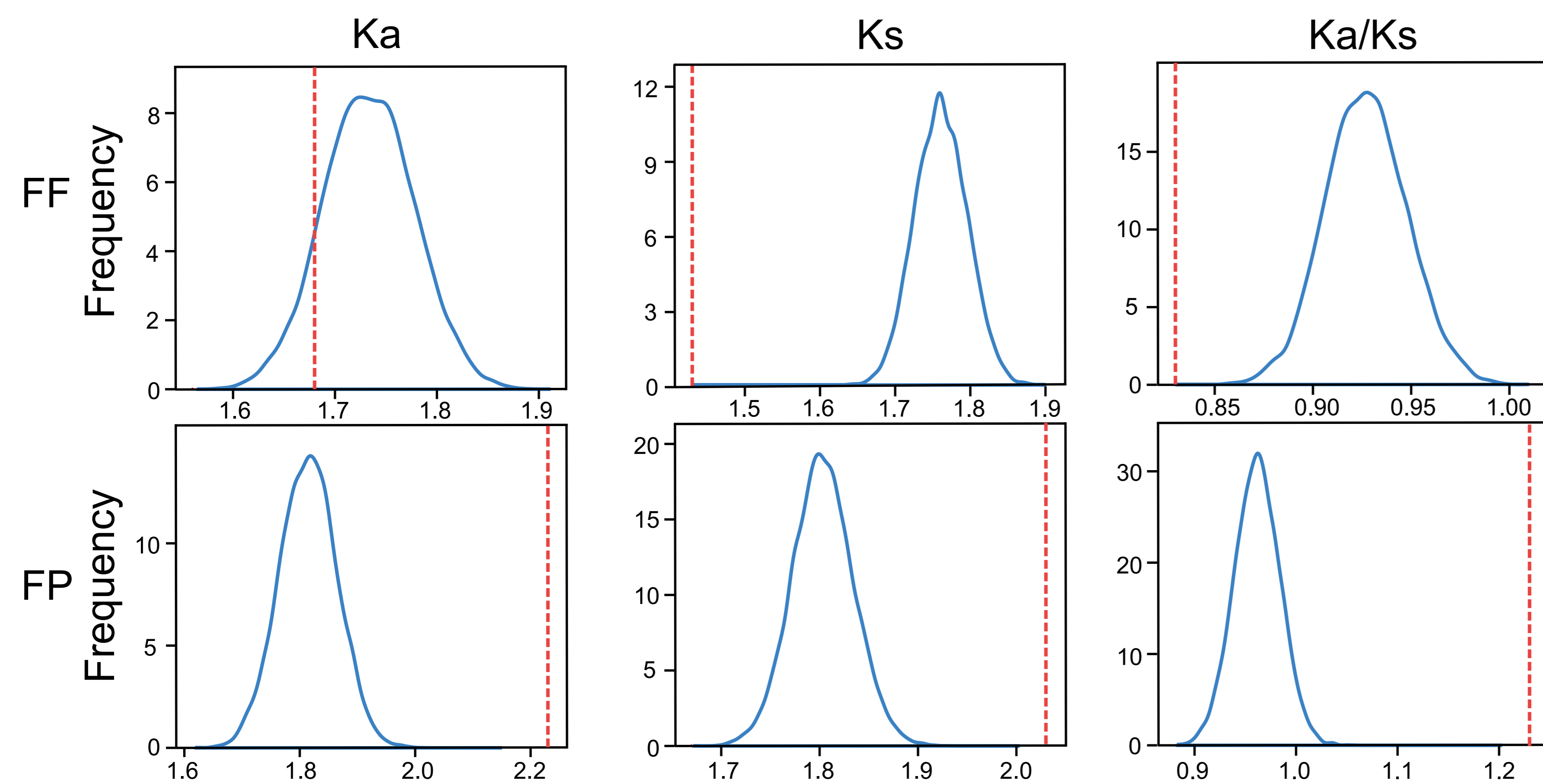

(D). Pr

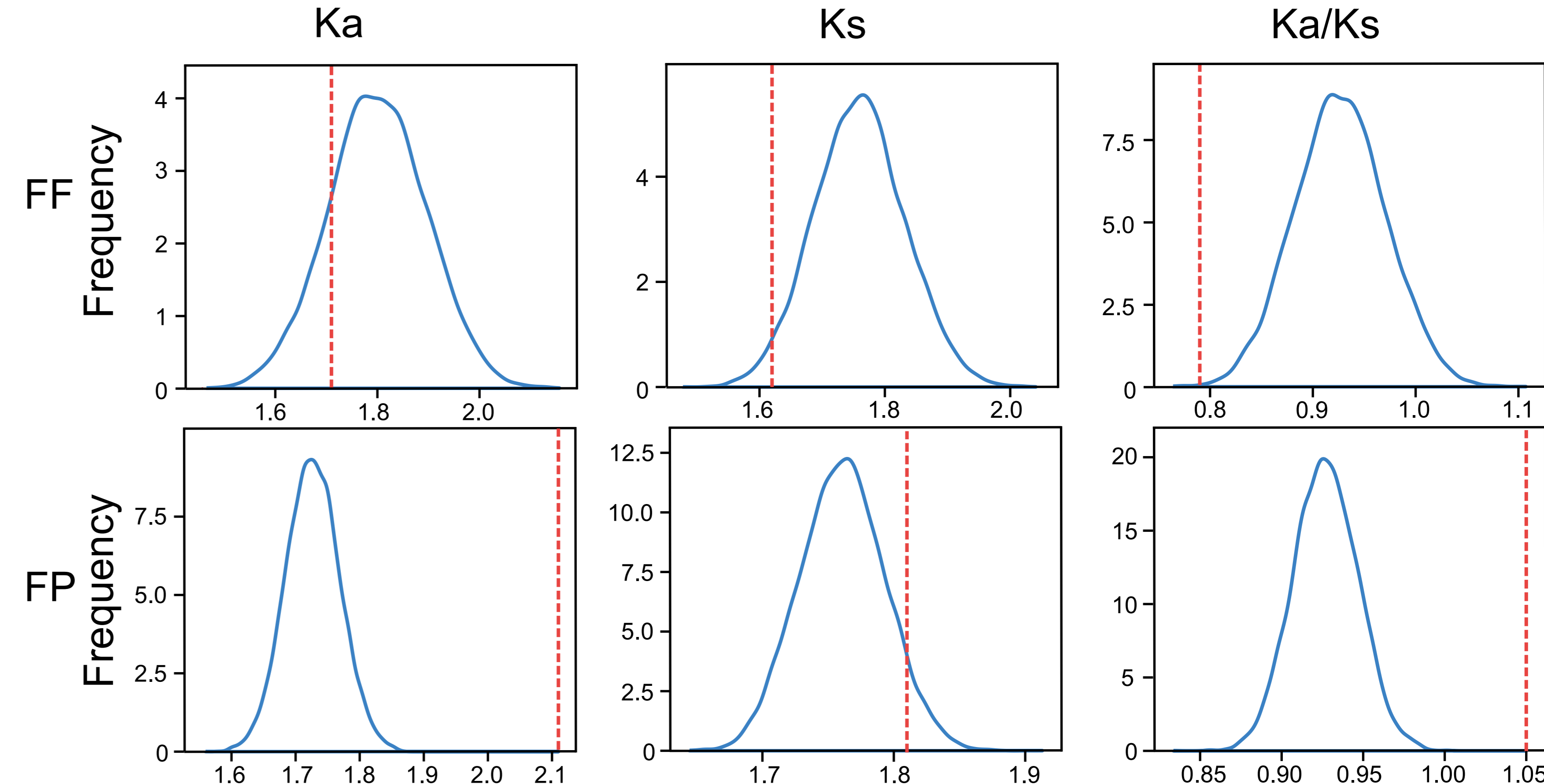

Supplement: Supplementary file 14 — Additional file 14: Figure S3. The frequency distributions of 10,000 repetitions of the randomized experiment for determining the Ka and Ks values, as well as the Ka/Ks ratio of FF and FP DEPs under four different types of stress. Navy blue corresponds to the randomized experiments, whereas the red dashed line corresponds to the real values. [file 12870_2020_2460_MOESM14_ESM.pdf]

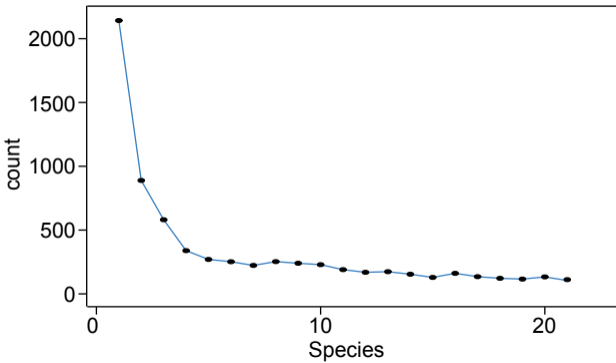

Supplement: Supplementary file 15 — Additional file 15: Figure S4. The inversely proportional correlations between species conservation and family size of the paralogous gene pairs. [file 12870_2020_2460_MOESM15_ESM.pdf]
